# Diversification and hybrid incompatibility in auto-pseudogamous species of *Mesorhabditis* nematodes

**DOI:** 10.1186/s12862-020-01665-w

**Published:** 2020-08-18

**Authors:** Caroline Launay, Marie-Anne Félix, Joris Dieng, Marie Delattre

**Affiliations:** 1 Laboratory of Biology and Modeling of the Cell, Ecole Normale Supérieure de Lyon, CNRS, Inserm, UCBL, 69007 Lyon, France; 2 IBENS, Département de Biologie, Ecole Normale Supérieure, CNRS, Inserm, PSL Research University, 75005 Paris, France

**Keywords:** Reproductive system, *Mesorhabditis* nematodes, Speciation, Asexuals, Species barrier, Hybrid incompatibility, Centrosomes, Auto-pseudogamy, Sperm size

## Abstract

**Background:**

Pseudogamy is a reproductive system in which females rely on the sperm of males to activate their oocytes, generally parasitizing males of other species, but do not use the sperm DNA. The nematode *Mesorhabditis belari* uses a specific form of pseudogamy, where females produce their own males as a source of sperm. Males develop from rare eggs with true fertilization, while females arise by gynogenesis. Males thus do not contribute their genome to the female offspring. Here, we explored the diversity of reproductive mode within the *Mesorhabditis* genus and addressed species barriers in pseudogamous species.

**Results:**

To this end, we established a collection of over 60 *Mesorhabditis* strains from soil and rotting vegetal matter. We found that males from pseudogamous species displayed a reduced size of their body, male tail and sperm cells compared to males of sexual *Mesorhabditis* species, as expected for males that face little competition. Using rDNA sequences and crosses, we could define 11 auto-pseudogamous biological species, with closely related species pairs and a possible single origin of pseudogamy in the *Mesorhabditis* genus. Most crosses between males and females of different species did not even produce female progeny. This surprising species barrier in pseudogamous egg activation was pre or postcopulatory depending on the species pair. In the latter case, when hybrid embryos were produced, most arrested before the first embryonic cell division. Hybrid incompatibility between auto-pseudogamous species was due to defective interaction between sperm and oocyte as well as defective reconstitution of zygotic centrosomes.

**Conclusions:**

We established a collection of sexual and pseudo-sexual species which offer an ideal framework to explore the origin and consequences of transition to asexuality. Our results demonstrate that speciation occurs in the pseudogamous state. Whereas genomic conflicts are responsible for hybrid incompatibility in sexual species, we here reveal that centrosomes constitute key organelles in the establishment of species barrier.

## Background

Sperm-dependent parthenogenesis, also called pseudogamy, is a reproductive strategy in which females use the sperm of males to activate their oocytes. Most known pseudogamous species parasitize males of sexual species, either closely related species (as an example, [1]) or more distantly related ones [2]. Foreign sperm is required for activation of embryonic development of the unreduced oocyte. However, in many cases the sperm DNA does not participate, and progeny develop solely from the maternal genome (i.e gynogenesis). In some species, sperm acceptance can occur sporadically, which leads to formation of polyploid individuals with important evolutionary consequences. This reproductive system, although rare, has been described in several plant and animal taxa, including vertebrates [3, 4]. In the nematode *Mesorhabditis belari*, a special type of pseudogamy is found, which we call auto-pseudogamy, where, as a source of sperm, females produce their own males at low frequency [5, 6]. Populations of *M. belari* are thus mainly composed of females but also of a lower frequency of males (9% in strain JU2817). These males are needed because their sperm trigger oocyte activation. Most eggs however do not use the sperm DNA after fertilization and develop into females by gynogenesis. In some eggs, the male DNA is used, and such amphimictic eggs only give rise to males. However, both asexual (females) and sexual (males) eggs are diploid. In oocytes that give rise to gynogenetic embryos, a single round of meiotic division is observed and a single polar body is formed (unreduced oocytes), thus compensating for the lack of the paternal haplome. In amphimictic embryos, two rounds of female meiotic division give rise to a haploid female genome (reduced oocytes), which then mixes with the haploid male DNA, producing a diploid male [5]. The males never further transmit their mother’s genes back to females. The interest in producing such males is to ensure that females will find available sperm to activate their oocytes. We previously showed that *M. belari* females are unable to mate with males from three other *Mesorhabditis* species, including two standard sexual species. *M. belari* is thus auto-pseudogamous, a reproductive strategy that we showed to be evolutionary stable [5]. The fact that amphimictic eggs always give rise to males is explained by the fact that although sex determination is through a XY system, an almost complete Y-bearing sperm drive occurs at fertilization [5].

Biological species are delineated on the basis of crosses between males and females giving rise to fertile offspring in both directions. Hybrid incompatibilities between sexual species most often rely on genetic conflicts between the divergent parental genomes (reviewed in [7]). For example, the lethality of *Drosophila simulans* and *D. mauritiana* hybrids is due to rapid evolution of DNA-binding homeobox domains [8], while hybrid embryos from crosses between diploid *Xenopus tropicalis* and tetraploid *X. laevis* die upon mis-segregation of some paternal chromosomes leading to a metabolic crisis [9]. With pseudogamy, the gynogenetic embryos do not use the sperm DNA so the mechanism of a block would be less obvious to explain.

The *Mesorhabditis* genus includes sexual species with standard sex ratio as well as species with a low proportion of males in nature [10, 11]. Like many nematode species, they have been classically described on the basis of morphology, but no crossing tests nor molecular tags have been used and the animals have not even been cultured.

Here we established a frozen culture collection of 66 strains of *Mesorhabditis* and use it to answer several pending questions: 1) Did pseudogamy arise once or repeatedly in the genus? 2) Did speciation occur in the pseudogamous state? 3) Can the sperm of males of other species be used at least to produce females by gynogenesis? 4) If not, at which stage does the species barrier block embryonic development? We identified 11 distinct auto-pseudogamous species that appear to form a single clade within the *Mesorhabditis* genus and explored the mechanisms of barrier between these species.

## Results

### Establishment of a collection of sexual and pseudogamous Mesorhabditis strains

**Fig. 1:**
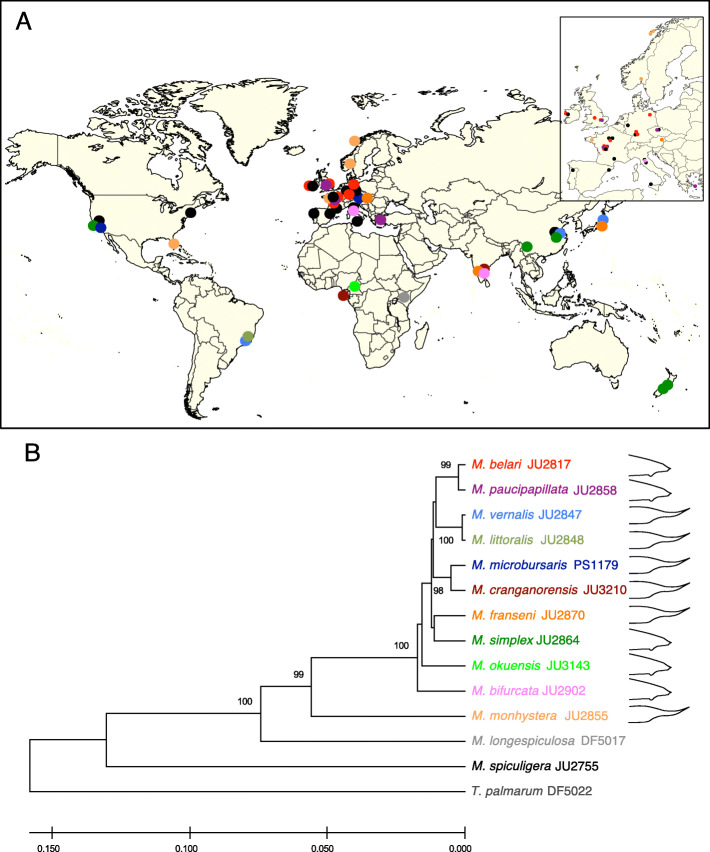
Geographic distribution and phylogenetic relationships of *Mesorhabditis* species. Sexual species are shown in black. Asexual species are color coded as in panel B. **a** The geographic origin of strains (as listed in Table S1) is shown as colored dots on a world map (under Creative Commons license), with an inset showing their distribution in Europe. **b** The phylogenetic relationships were deduced from analysis of 28S and ITS2 rDNA sequences. Only those bootstrap values (100 replicates) higher than 95 are shown. The male tail morphology of species, classified as short or long tail, is shown as schematic drawings

We brought in culture from soil and rotting vegetation 66 new *Mesorhabditis* isofemale strains from all 5 continents (Table S1, Fig. 1). We could freeze the strains and revive them by thawing, thus establishing a stable frozen collection (see Methods). In this study, we also analyzed the previously characterized sexual strains *M. spiculigera* AF72 and JU764 and *M. longespiculosa* DF5017, as well as the pseudogamous strains *M. belari* JU2817 and *M. microbursaris* PS1179.

We first categorized these strains as sexual (*n* = 18) because of a 1:1 sex ratio or as pseudogamous strains (*n* = 48) because of a highly biased sex ratio (Supplementary Text, Table S1).

### Diversity and monophyly of pseudogamous strains

We obtained molecular tags in ribosomal DNA for each strain and used these sequences in two ways: 1) to group species based on similarity (Table S2); 2) to explore possible phylogenetic relationships among them based on these sequences (Figure S1). For the first aim, we identified clear groups of pseudogamous strains with identical tags within the pseudogamous strains (pair-wise distance = 0). For example, we found 12 strains identical to *M. belari* JU2817, suggesting they are all strains of the same species. These strains were distant from *M. microbursaris* PS1179 (pair-wise distance > 0.2) and we did not find other strains identical to PS1179. We found 7 other groups of identical sequences, suggesting the existence of at least 9 pseudogamous species. Within the sexual strains, none of the strains shared an identical sequence. However, all new strains were close to *M. spiculigera* JU764 (pair-wise distance < 0.1) while *M. longespiculosa* DF5017 was far from all the others (pair-wise distance > 0.1). This result suggests that our sampling of sexual strains identified only new strains of *M. spiculigera*. Regarding the second aim, we found that using this small set of sequences, the monophyly of pseudogamous strains is highly supported (99% of bootstraps). *M. longespiculosa* is the closest sexual species, sister to the group of pseudogamous species. Within the pseudogamous clade, a deep branch separates a subclade including JU2855 and 5 other strains from a larger subclade including *M. belari* JU2817.

### Biological species can be defined among pseudogamous strains using crosses

We next wondered whether species barriers existed between pseudogamous strains, even for embryos that developed gynogenetically without the sperm DNA. We thus established crosses between pairs of pseudogamous strains (Table 1 and Table S3). We prioritized crosses based on tag sequences. We found that strains with similar molecular tags are able to produce F2 progeny in both directions of the cross, while strains with distant molecular tags, for instance *M. belari* strains and strains from the JU2855 group, do not produce cross-progeny, as expected for biological species. Thus, despite the same pseudogamous mode of reproduction, species barriers are established and the biological species concept can be applied. A few exceptions to the sharp grouping into biological species are found and are detailed below.

**Table 1:**
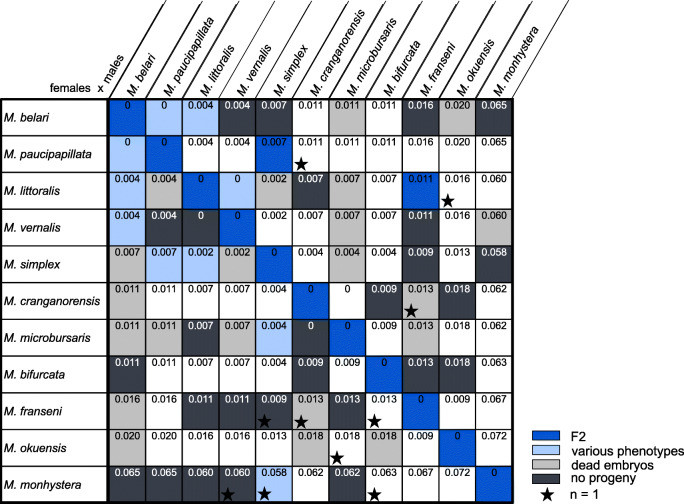
Crosses between *Mesorhabditis* pseudogamous species. Summary of the crosses shown in Table S3. Cross outcomes are color-coded: blue for viable F2 progenies, black for absence of embryo production, light grey for production of dead embryos and light blue for mixed results, including the production of sterile F1s. * only one strain per species was tested. For each pair of species, the pair-wise distance between strains selected for Fig. 1 is shown.

We find a range of incompatibilities between species, from the absence of eggs to the production of dead embryos only, or the production of viable F1s in only one cross direction. The latter type of results may reflect more recent species isolation. Based on these criteria, we could distinguish 11 pseudogamous species: 9 species corresponding to the 9 groups of strains with a pair-wise distance of 0, 2 species with a single representative strain for each (JU2848 and PS1179) and 1 species with 2 strains (JU2902 and JU3211).

Next, we assigned names to each of these species. Many *Mesorhabditis* species with a low fraction of males had been previously described [12, 13]. We compared our cultures to previous morphological descriptions, in particular those of the male tail when available (Supplementary Text and Figure S2). We matched 7 of them to a previously described species: *M. paucipapillata* (JU2858 group), *M. franseni* (JU2870 group), *M. simplex* (JU2864 group)*, M. vernalis* (JU2847 group), *M. littoralis* (JU2848), *M. cranganorensis* (JU3172 group) and *M. monhystera* (JU2855 group). For the two remaining groups to which we could not assign existing species names, we described here two new species as *M. bifurcata* n. sp. for the species including JU2902 and JU3174 and *M. okuensis* n. sp. for JU3143, JU3147 and JU3148 (see Supplementary Text for the species description).

In the *M. monhystera* subclade, JU3162 reproduces well with JU2855, despite their large molecular distance, and we thus consider it as a *M. monhystera* strain (Table S2, Table S3). JU3162 is from North America, while the other five strains are from Europe, perhaps explaining the molecular divergence.

Within the larger pseudogamous subclade, we find biological species barriers even at low molecular divergence, for example between 3 pairs of sister species: *M. belari / M. paucipapillata*, *M. vernalis / M. littoralis*, *M. microbursaris / M. cranganorensis* (Fig. 1). As with many biological species, the barrier may be imperfect. JU3130, which belongs to the group of *M. paucipapillata* strains from tag sequencing, is able to reproduce with both *M. belari* and *M. paucipapillata* (Table S3). Other strains of these two groups are also able to produce viable F1s in rare cases (for instance JU2890 and JU2817). JU3003 however appears from the molecular tags to be outside of the *M. paucipapillata / M. belari* pair; however, it reproduces well with *M. paucipapillata* JU3130 but not *M. belari* JU2817 (Table S3), suggesting that it may be classified as another *M. paucipapillata* strain. Overall, these results suggest a recent isolation between this pair of species.

Some crosses gave less clearcut results. For instance, while the majority of strain pairs from *M. belari* and *M. vernalis* are unable to produce eggs, a few strain pairs sometimes produce viable F1s. The same situation was found for *M. belari* and *M. monhystera* strains (Table S3).

Concerning the new sexual strains, we performed crosses with 10 of them (Table S3). We chose strains with small and large pair-wise distance with AF72 or JU764. They all yielded F2s when mated with *M. spiculigera* AF72 or JU764. Moreover, they had seemingly the same male tail morphology than AF72 and JU764, strongly suggesting they are all strains of *M. spiculigera* (data not shown). With, in addition a single strain of *M. longespiculosa* DF5017, our collection thus only includes two sexual species.

### Females derive from gynogenesis and males from rare amphimixis in all tested pseudogamous Mesorhabditis species

**Fig. 2:**
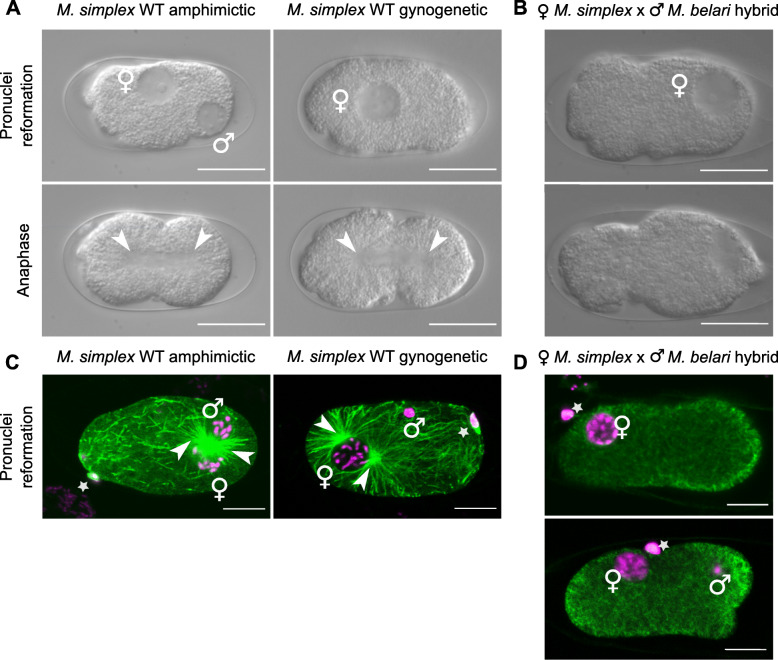
Phenotypes of wild-type and hybrid embryos. **a-b** Still images from DIC recordings of representative amphimictic and gynogenetic embryos in wild-type *M. simplex* JU2864 (**a**) or in a gynogenetic hybrid embryo from a cross between *M. simplex* JU2864 females and *M. belari* JU2817 males (**b**). Embryos before and during the first cell division are shown in the upper and lower panels, respectively. **c-d** Fixed specimen during the reformation of pronuclei in wild-type *M. simplex* (**c**) and hybrid embryos (**d**). Microtubules are in green and DNA in magenta. The second polar body of the amphimictic embryo is on a different focal plane and not visible here. In (**d**), the sperm DNA has entered the embryo shown on the lower panel but not that on the upper panel. The polar body is indicated with a star and the centrosomes with arrows. Scale bar is 10 μm

By analogy with *M. belari* JU2817, we suspected that species showing a biased sex ratio reproduced by pseudogamy, i.e. produced females by gynogenesis. To confirm this hypothesis, we analyzed representative strains of 5 other species with biased sex ratio: *M. paucipapillata, M. simplex*, *M. okuensis*, *M. bifurcata* and *M. monhystera*. First, we recorded embryos after fertilization by time-lapse DIC microscopy (Movies S1, S2, S3 and S4). As in *M. belari* JU2817, the majority of embryos developed by gynogenesis (Movies S1 and S3) with no appearance of a paternal pronucleus after fertilization (Fig. 2a). Cytological observations confirmed that the male DNA stayed condensed after fertilization and was set aside (Fig. 2c). For all strains we recovered gynogenetic embryos and found that they always gave rise to females (Table S5). Few embryos arose from amphimixis (Movies S2, S4), and all gave rise to males as in *M. belari* JU2817 (Fig. 2, Table S5). Thus, these species also produce females by gynogenesis and males by amphimixis. From these results we confirmed that *Mesorhabditis* species that display a biased sex ratio reproduce by auto-pseudogamy and that auto-pseudogamy is widespread within the *Mesorhabditis* genus.

### Variation in sex ratio

Using a game theory model to explain the long-term maintenance of males whose genes are not passed on to their female offspring, we previously showed that the production of males below 15% was sustainable in the long term [5].

**Fig. 3:**
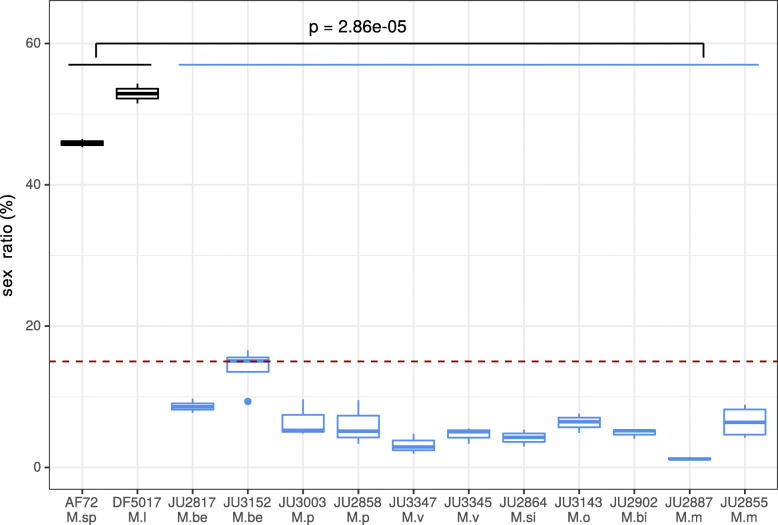
Variation in the sex ratio between *Mesorhabditis* strains. Boxplot representation of the sex ratio for 2 sexual species and 11 strains of auto-pseudogamous *Mesorhabditis* species, corresponding to 6 species. For each measurement, between 200 and 600 individuals were counted, 2 to 4 times independently depending on the strain (see corresponding values in Table S4). The sex ratio of asexual species is statistically different than those of the sexual species (GLMM model). The red dotted line corresponds to the highest sex ratio predicted by a game theory model for auto-pseudogamous species

We measured the sex ratio of 7 pseudogamous species, including 2 strains for some species, as well as 2 sexual strains. The sex ratio of the pseudogamous species were statistically different from those of the sexual species (pvalue = 2.86e-05, using a Generalized Linear Mixed Model (GLMM), Fig. 3, Table S4). Among the pseudogamous species, we found variation in male proportion ranging from 1.26% for *M. monhystera* JU2887 to 13.97% for *M. belari* JU3152. We also found intraspecies variation. We concluded that the proportion of males and thus of amphimictic eggs vary between and within pseudogamous species under identical laboratory conditions. The measured values, all below 15%, are compatible with the game theory model. The variation between strains could reflect evolutionary changes in the number of gametes produced, but also in migration rates or mating preferences [5].

### Morphological reduction of males in pseudogamous species

**Fig. 4:**
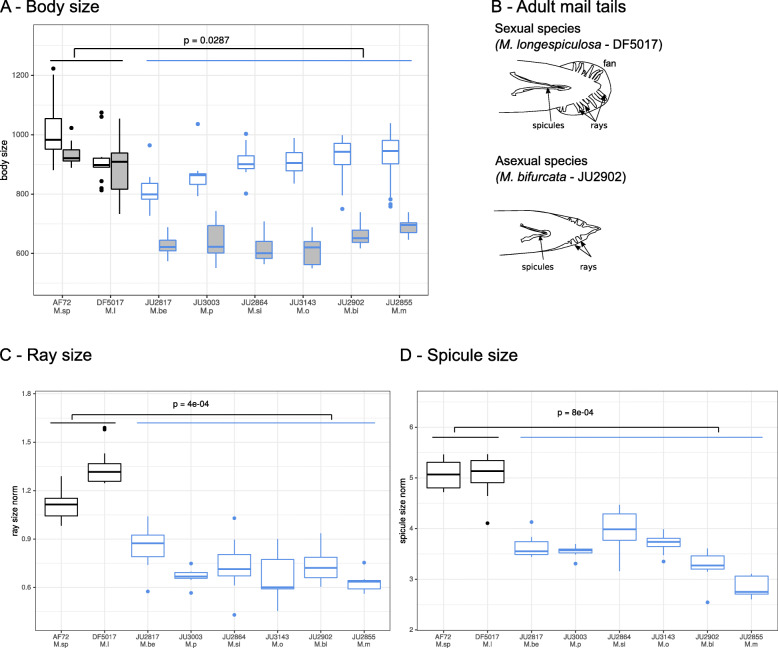
Reduction of male body size and reproductive apparatus in auto-pseudogamous *Mesorhabditis* species. The raw data are in Table S4. For each measure, between 8 and 20 individuals were analyzed. Sexual species are shown in black and pseudogamous species are in blue. *M. sp: M. spiculigera* AF72, *M. l: M. longespiculosa* DF5017, *M. be: M. belari* JU2817, *M. p: M. paucipapillata* JU3003, *M. si: M. simplex* JU2864, *M. o: M. okuensis* JU3143, *M. bi: M. bifurcata* JU2902, *M. m: M. monhystera* JU2855. **a** Boxplot of body size for females (in white) and males (in grey) for each strain. **b** Drawing of the male tail, to scale, in a sexual and a pseudogamous strain, in ventral view. **c** Boxplot of the length of the longest rays as a proportion of male body size for each strain. **d** Boxplot of spicule size shown as a proportion of male body size for each strain. A GLMM model has been used to test the statistical difference in morphological traits (**a**, **c**, **d**) for pseudogamous species compared to sexual species

We noticed that the sexual dimorphism in body size was more pronounced for the pseudogamous than for the sexual species. We measured body size for males and females from species of both groups. Using a GLMM model we tested the effect of the reproductive mode and the sex, as well as the interaction between them on body size. We found that each term was significant (pvalue < 0.05, pvalue< 0.001, pvalue< 0.001, respectively). We concluded that the body size of males from pseudogamous species was systematically smaller than those from sexual species (Fig. 4, Table S4).

We also found that in agreement with prior observations [13, 14], the reproductive apparatus of males was reduced in auto-pseudogamous species compared to sexual species. Proportionally to body size, spicule size and ray sizes were in general twice smaller in pseudogamous males compared to sexual males (GLMM model, pvalue = 8e-04 and 4e-04, respectively, Fig. 4). For some strains, for instance *M. simplex* JU2864, rays were so short that they were difficult to identify [11]. The number of rays was also reduced and more variable between individuals in pseudogamous males (Figure S2).

The length of the male tail varied among the pseudogamous species. Sexual *Mesorhabditis* species have a rounded fan, while several pseudogamous strains show a longer tail that can be considered as a lack of retraction of the L4 larva tail at the adult molt [15]. For example, the male tail is long in *M. monhystera,* the species pairs *M. vernalis / M. littoralis* and *M. microbursaris / M. cranganorensis* and short in the species pair *M. belari* / *M. paucipapillata*, as well as in *M. simplex* and the two new species (Table S1, Figure S2). In agreement with a versatile trait, within strain variation has also been observed in *M. vernalis* JU2847, where males may display tails of various lengths (Figure S2I). The lack of resolution of our phylogeny does not allow us to conclude whether the ancestor of all pseudogamous species had a short or long tail (Fig. 1).

**Fig. 5:**
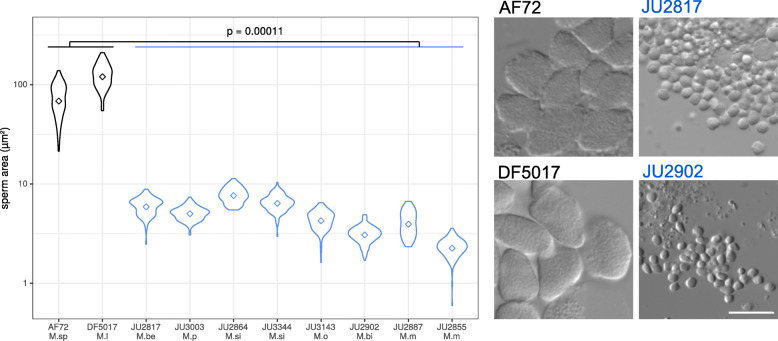
Drastic reduction of sperm area in pseudogamous *Mesorhabditis* species. The raw data are in Table S4. Sexual species are shown in black and pseudogamous species are in blue. *M. sp: M. spiculigera* AF72, *M. l: M. longespiculosa* DF5017, *M. be: M. belari* JU2817, *M. p: M. paucipapillata* JU3003, *M. si: M. simplex* JU2864, *M. o: M. okuensis* JU3143, *M. bi: M. bifurcata* JU2902, *M. m: M. monhystera* JU2855. **a** Violin boxplot of sperm area in μm2 for sexual and asexual *Mesorhabditis* strains. A GLMM model has been used to show the effect of the reproductive mode on sperm area. **b** Representative DIC pictures of sperm cells. Scale bar is 10 μm

Finally, we found a dramatic difference in sperm cell area between the two groups. The mean area of sperm was 73 and 124 μm2 in the sexual species *M.spiculigera* and *M.longespiculosa*, respectively, while sperm area ranged from 2.3 to 7 μm2 in the auto-pseudogamous species (GLMM model, pvalue = 1.1e-04, Fig. 5, Table S4).

In summary, all auto-pseudogamous species were characterized by a very low frequency of males, whose body size, reproductive apparatus and sperm size are extremely reduced compared to males of the sexual *Mesorhabditis* species. In addition, we found morphological differences among auto-pseudogamous species, as well as variation in sex ratio.

### Absence of egg production in auto-pseudogamous hybrid crosses is due to precopulatory isolation

The reproductive isolation that we uncovered among auto-pseudogamous species begs the question of the mechanism for this incompatibility. The sperm DNA is not used in gynogenetic embryos so chromosome content or paternal genome expression are unlikely its cause. By performing crosses between species (Table S3), we found signs of precopulatory isolation (no embryo production), while some crosses yielded dead embryos. We further analysed these crosses.

First, we tested whether the absence of laid embryos was due to a precopulatory (no mating and sperm transfer) or postcopulatory barrier (no egg/sperm recognition, i.e. gametic isolation). We focused on crosses between *M. belari* and *M. monhystera*. Males were bath in Mitotracker Red, and after crosses, we searched for red sperm in the spermatheca of females. In control experiments between males and females of the same strain, 13 out of 15 females showed a red-labeled spermatheca after 48 h of mating, indicating sperm transfer. In contrast, none of the 40 *M. belari* JU2817 females crossed with labelled *M. monhystera* JU2855 males showed a red spermatheca (Figure S3), indicating the absence of sperm transfer. From this result, we conclude that a precopulatory barrier with no sperm transfer exists between *M. belari* and *M. monhystera*.

### Production of dead embryos in auto-pseudogamous hybrid crosses is due to sperm/oocyte incompatibilities or defective reconstitution of paternal centrosomes

Next, we analysed crosses that produce dead embryos. We first concentrated on crosses involving *M.simplex* JU2864, which gave rise to dead embryos when crossed with males of several other species (Table 1 and Table S1). When crossed with males of *M .belari* JU2817, *M. simplex* JU2864 produced many embryos, 95.5% of which died (*n* = 2321, Table 2). They also laid many unfertilized eggs, clearly recognizable by their dark color and absence of eggshell. The *M. simplex* JU2864 strain displayed only 2.4% of embryonic lethality (11/458 F1 progeny). Moreover, virgin JU2864 females (*n* = 70) did not produce any eggs or unfertilized oocytes when isolated, indicating that the production of dead eggs in the hybrid crosses is induced by the presence of foreign sperm in the female spermatheca. We then recorded the hybrid embryos by time-lapse DIC microscopy. While wild-type embryos are very resistant to mounting between slide and coverslip, we found that 13 out of 30 hybrid embryos exploded during mounting (Movie S5). By analogy with *C. elegans*, we suspected that the eggshell of these hybrid embryos was improperly formed in the uterus, leading to osmotic shock and overall fragility [16]. Among the 17 remaining intact embryos, 4 displayed an irregular egg shape, likely due to improper egg shell formation in the hybrids. These 17 embryos initially developed normally until nuclear envelope breakdown: meiosis resumed, the female pronucleus reformed and migrated within the cell. One embryo was amphimictic and 16 were gynogenetic. For the amphimictic embryo and 14 gynogenetic embryos, the first mitotic spindle did not form and the cell did not divide. Embryos were blocked for hours at this stage before eventually dying (Movie S6 and Fig. 2b). This cell cycle arrest was not induced by the slide mounting procedure itself because eggs laid on plates were also blocked at the 1-cell stage. The two remaining embryos however were able to divide and we followed 3 successive cell divisions. We further showed that this cell cycle arrest was not specific to this *M. simplex* strain by observing the same phenotypes in hybrids between *M. simplex* JU3344 females and *M. belari* JU2817 males (data not shown). Last, we showed that the hybrid embryos produced by other species pairs (*M .belari* JU2817 and *M. okuensis* JU3147 (*n* = 6) or *M. belari* JU2817 and *M. microbursaris* PS1179 (n = 6)) also died during the first cell cycle (Figure S4). Thus, the vast majority of hybrid embryos were arrested at the one-cell stage because they could not divide and/or because the eggshell was improperly formed. We propose that this early zygotic block is a general landmark of hybrid incompatibility in auto-pseudogamous species. For the few hybrid escapers that were observed, we hypothesized that cell divisions eventually stopped for most of those embryos as well, as only 0.47% of the total embryos produced did hatch.

**Table 2:** Analysis of hybrid crosses

Crosses		Eggs	F1 female	F1 male	Hatching rate (%)
Females	Males				
*M. belari* JU2817	*M. simplex* JU2864	0	0	0	0
*M. simplex* JU2864	*M. belari* JU2817	2321	10	1	0.47
*M. paucipapillata* JU2858	*M. belari* JU2817	few	0	0	0
*M. belari* JU2817	*M. paucipapillata* JU2858	172	88	17	61
F1 hybrid JU2817JU2858	*M. belari* JU2817	81	71	10	100
F1 hybrid JU2817/JU2858	*M. paucipapillata* JU2858	79	25	9	39
*M. belari* JU2817	F1 hybrid JU2817/JU2858	0	0	0	0
*M. paucipapillata* JU2858	F1 hybrid JU2817/JU2858	32	24	3	84

To determine whether the sperm of *M. belari* JU2817 had entered the oocyte of *M. simplex* JU2864 females, we observed the state of the DNA, cytoskeleton and centrosomes in hybrid embryos. Although most hybrid embryos were damaged by the fixation procedure (most likely due to their fragility), we were able to analyse 21 embryos. All of them showed polar bodies, demonstrating that female meiosis had resumed. The size and position of the female pronucleus as well as the condensation of its DNA varied between embryos, reflecting that these embryos were at different stages in the first cell cycle. In agreement with the phenotypes observed by DIC, the majority of embryos had not divided (*n* = 18) and three embryos had reached the 4-cell stage or the 8-cell stage (Fig. 2d). Among the 18 embryos blocked during the first cell cycle, 9 embryos had no sperm DNA inside the cell and no microtubule asters. Interestingly, one of them had two polar bodies, suggesting that it was engaged in amphimictic development. For the other 9 embryos, we found a single polar body and saw condensed sperm DNA inside the embryo, as seen in wild-type gynogenetic embryos. However, we could not detect microtubule asters in any of these embryos, even in the five embryos that had reached S phase, a stage at which asters are very large in wild-type embryos (Fig. 2c). Hence, in the hybrid embryos, female meiosis resumes, and the first embryonic cell cycle initiates, even in cases where sperm does not enter. Importantly, even when a sperm cell does penetrate the oocyte, its centrosomes do not reform, except for a few escapers (3 out of 21). From these results, we propose that embryonic lethality is here due to the absence of centrosomes, which prevents the formation of a spindle and embryonic cell divisions. However, some egg activation (meiosis) occurred by contact with the foreign sperm.

### Analysis of postzygotic isolation in auto-pseudogamous hybrid crosses

Last, we analysed the species pairs that produced viable F1s in a single direction of the cross. Although we could sometimes detect a handful of F2 larvae, such crosses never yielded a live population. Importantly, we systematically found F1 males. This allowed us to confirm that the low production of F2 progeny was due to the sterility of F1 individuals and not to the absence of males in the F1 progeny (Table S3). This result also showed that, unlike for classical pseudogamous species, pseudogamous *Mesorhabditis* females do not parasitize the males of other *Mesorhabditis* species for the production of females. According to Haldane’s rule, sterile F1 individuals from a hybrid cross are more likely to be of the heterogametic sex, here males [17]. We thus tested whether F1 hybrids of either sex were fertile when backcrosses to their parental strains (Table 2). From a cross of virgin *M. belari* JU2817 females with *M. paucipapillata* JU2858 males, we isolated F1 hybrid virgin females and crossed them with males from the parental strains. Hybrid females produced 100% viable progeny when mated with *M. belari* JU2817 males, while only ca. 40% of the progeny was viable when mated with *M. paucipapillata* males (Table 2). Because hybrid females inherited only the genome of their mother, here that of *M. belari*, a reduced reproductive success with *M. paucipapillata* was expected. When hybrid males were crossed with *M. belari* virgin females, no eggs were produced. In contrast, mating of these males with virgin *M. paucipapillata* females produced eggs that were 100% viable. Thus, hybrid males are not sterile but they cannot reproduce with *M. belari* females. As the F1 females are genetically identical to *M. belari* females, mating between F1 hybrids siblings cannot produce a progeny.

## Discussion

### A culture collection of diverse Mesorhabditis strains

We here established a culture collection of over 60 *Mesorhabditis* strains, available for further studies. By sampling soil and rotting vegetal matter, especially leaf litter, we found across the world many pseudogamous strains as well as new strains of a single sexual species, *M. spiculigera*, previously described to be cosmopolitan [10]. Unfortunately, we could not isolate the other sexual species that were previously defined morphologically, also initially found in soil and rotting vegetation [10, 12]. One likely explanation is that we did not sample sufficiently and/or the right substrates [14]. Another possible partial explanation is that *M. spiculigera* displays phenotypic polymorphisms that have been used to define morphological species.

Amazingly, unlike other asexual species, the pseudogamous reproductive system of *Mesorhabditis* allowed us to perform crosses and define different biological species. The evolutionary and biological implications of this crucial finding are further discussed below. In terms of systematics, we identified at least 11 distinct pseudogamous species. Although our sampling is limited and biased towards Europe, some species were found on several continents while others seemed restricted to one continent. For instance, we found *M. belari* and *M. paucipapillata* only in Europe, while *M. simplex* is present in America, Asia and Oceania.

This collection of strains and species offers an ideal framework to explore the origin and consequences of pseudogamy within the *Mesorhabditis* genus. Based on cladistic analysis of phenotypic traits, Sudhaus 1976 (translated in [11]) proposed that, although pseudogamy itself is derived, the *Monhystera* group (the pseudogamous species) branches basally within *Mesorhabditis*, which is not confirmed here. Phylogeny based on more markers or whole-genome sequencing will further be required to unambiguously conclude on the relationship between these species. The inclusion of more sexual species may be also required to confirm that pseudogamy emerged once within the *Mesorhabditis* genus, as suggested here. Nevertheless, our analysis clearly indicates that speciation occurred repeatedly in the pseudogamous state.

### Evolutionary consequence of the pseudogamous state on the male reproductive system

When compared to males of the closest sexual species, males of all pseudogamous species were small and displayed a reduced copulatory apparatus, in particular small sperm cells (below 6 μm2). These morphological differences are likely explained by the reduced male-male competition in species composed of more than 85% females [18]. By contrast, the very large size of sperm cells of *Mesorhabditis* sexual species suggests that they face strong male/male competition. Interestingly, within the *Caenorhabditis* genus, sperm size of sexual species is often in the range of 20–50 μm2. Nevertheless, species with giant sperm, above 100 μm2 emerged at least 4 times independently [19]. Whether the large sperm of *Mesorhabditis* sexual nematodes (ca. 100 μm2) is usual in this group of species or whether it reflects exaggerated competition between males in the two tested species remains an open question.

### Pseudogamous species barrier for both male and female progeny

As mentioned above, pseudogamous species in other phyla use males of other species to fertilize their oocytes and the mating do not produce males. One could expect that crosses between closely related *Mesorhabditis* species could yield viable F1 females and that only the production of sexual males by true fertilization is perturbed. To our surprise, for most crosses between strains we obtained only two categories of results: i) fully viable F2s, interpreted as crosses between strains of the same species, ii) no embryos or lethal embryos, interpreted as crosses between distinct species. In the few cases where crosses yielded viable but sterile F1s, we systematically also identified viable males, which we interpreted as crosses between close species. From this study, we thus concluded that the *Mesorhabditis* pseudogamous females in our collection are not able to produce gynogenetic females with males of other species. Instead, conspecific mating is preferred in these exclusively auto-pseudogamous species.

Nevertheless, few crosses between strains that are close in terms of rDNA sequence gave intermediate results. This was particularly true for *M. belari* and *M. paucipapillata* strains or *M. belari* and *M. vernalis*. In particular, we found that JU3130 was able to reproduce equally well with *M. belari* JU2817 and *M. paucipapillata* JU2858. Such an example may be precious for further studies of speciation in this group as it could indicate cases of either co-dominant alleles or paralogs involved in the incompatibility.

### Speciation in the pseudogamous state: evolutionary context

Whether the rate of speciation is similar for sexual and asexual biological species is still an open question in evolutionary biology [20]. Solving this question requires collections of sexual and asexual strains and the ability to delineate biological species for asexuals, which is by definition impossible - except in particular cases such as described here. Although analysis of molecular divergence in some clades has led to the identification of distinct species and analysis of speciation rates within asexual Rotifers [21] or Oribatid mites [22], empirical data are clearly missing. Our analysis of pair-wise distance between *Mesorhabditis* strains revealed a trend, similar to what had been observed for Rotifers [21], were clusters of closely related individuals (distance close to 0) are separated from other clusters in asexuals. By contrast pair-wise distance in rDNA among sexual strains was much higher (Figure S1). The possibility to use the biological species definition in *Mesorhabditis* nematodes allowed us to unambiguously define species, thus offering an ideal situation to explore speciation in the asexual and sexual regimes. Although more extended analysis of genetic distance will be required, our results suggest that little genetic variation is sufficient to transition to a new species in this asexual regime, while intraspecific genetic diversity accumulates with sexuality and recombination.

Pseudogamous species have also been shown to be the driver of speciation for closely related sexual species [23]. Indeed, pseudogamous species often arise from a hybridization event between sexual species [3]. As such, they contribute to reproductive isolation by preventing gene transfer as sterile hybrids would do. In this context, it will be crucial to determine whether speudogamous *Mesorhabditis* derive from an ancient hybridization event.

Using a game theory model, we previously showed that the production of rare males whose genes were not transmitted to the female offspring was an evolutionary stable system provided that males would preferentially mate with their sisters. This mating bias may derive either from a low migration rate hence physical proximity between siblings or from an active mate choice. Indeed, the only stable strategy for a female to produce males is to ensure that its daughters will be fertilized. If migration rates were infinite, the female strategy of producing no males could win and lead to population extinction [5]. The preferential sib mating that is required for the evolutionary stable strategy is compatible with the evolution of mating bias and incompatibility barriers. If the species barrier only concerned a classical genetic incompatibility between the two parental genomes in males, only females would be produced in crosses between the two species. The evolutionary stable system would be disrupted and one species would parasitize and be fully dependent on the other. We therefore hypothesize that *Mesorhabditis* nematodes are strict auto-pseudogamous species and that pseudogamous species (sperm parasitism) are unlikely to coexist. Nevertheless, it must be noted that we would have missed a species that would reproduce by pseudogamy using parasitism since we brought them in culture by isolating a single female, and only kept lines when the isolated females produced males in their progeny, allowing for further maintenance (see Material and Methods).

### Incompatibility mechanisms in the pseudogamous state

As for sexual species, we found all range of incompatibility barriers between auto-pseudogamous *Mesorhabditis* species. Similar to previous work performed on reproductive isolation in sexual species [24–26], our results suggest a relationship between the genetic distance between species and the isolation mechanism (see Table 1). Indeed, the incompatibility mechanism between the distant species *M. belari* and *M. monhystera* is pre-copulatory, at least acting prior to sperm transfer. On the other side, the species barrier between the closest species *M. belari* and *M. paucipapillata* is due to F1 sterility. In between, some species pairs were able to mate and produce activated embryos, indicating that copulation and sperm-oocyte recognition were possible. Nevertheless, the vast majority of hybrid embryos died, most of them because of defects during the first cell cycle. To formally conclude on these results, more accurate measurement of genetic distances (i.e on many more markers or whole genome sequencing) will be required.

In *C. elegans* nematodes, the contributions of the sperm for the different steps of zygote formation have been clearly identified [27]. Sperm cells are stored in the spermatheca after mating. First, sperm cells secrete the Major Sperm Proteins which trigger oocyte activation and passage through the spermatheca. Second, the physical contact between sperm and oocyte activates three independent events: i) the formation of the eggshell and permeability barrier, two outer coats that are essential for zygote survival, ii) the completion of female meiotic divisions and iii) sperm penetration into the oocyte. Third, once the sperm has entered the oocyte, it provides DNA and a pair of centrioles. The sperm DNA is facultative, as anucleate sperm can sustain the early zygotic divisions in *C. elegans* [28]. However, the centrioles are necessary for development: at fertilization, the paternally provided centrioles recruit the pericentriolar proteins present in the oocyte cytoplasm, which leads to the reconstitution of the first zygotic centrosomes [29, 30]. In *C. elegans*, in the absence of functional centrosomes, the cell is unable to form a mitotic spindle and zygotic cell divisions cannot proceed [31, 32]. We found that the death of a large proportion of hybrid *Mesorhabditis* eggs was due to their explosion soon after they were released from the spermatheca, most likely due to a defective eggshell and permeability barrier [33]. The eggs that did not explode progressed through meiosis regardless of the presence of the sperm DNA inside the cell, suggesting that physical contact without gamete fusion may be sufficient to trigger the oocyte meiotic divisions. Whether a sperm DNA had entered the cell or not, all embryos were lacking functional centrosomes (no microtubule asters were visible) and consequently were unable to form the first mitotic spindle. By analogy with what is known in *C. elegans* we thus hypothesize than in the hybrid crosses, the foreign sperm is i) sufficient to trigger ovulation, ii) competent to trigger meiotic divisions, iii) not always competent to penetrate inside the oocyte, iv) always incompetent to provide material to build microtubule-nucleating centrosomes. Whether the centrosomes are not formed in the hybrids where gamete fusion occurred because the paternal centrioles of the other species are not able to recruit the maternal pericentriolar proteins or because they are actively inhibited or degraded by the oocyte remains to be explored.

The trigger for the interspecific recognition could be located on the sperm surface, sperm DNA/chromatin, centrioles, mitochondria, small RNAs, proteins or any fast-varying biochemical components. Given the bias whereby only Y-bearing sperm allows development of embryos (gynogenetic and amphimictic) in conspecific crosses of *M. belari* [5], it is tempting to speculate that the incompatibility block may have common mechanisms with the X-bearing sperm block.

Interestingly, we found that hybrid embryos between *M. simplex* and *M. belari* could adopt a gynogenetic (one polar body) or an amphimictic fate (two polar bodies due to two rounds of female meiotic divisions) regardless of the presence of the sperm DNA inside the oocyte. This finding suggests that maternal factors rather than paternal factors are responsible for the decision between an amphimictic and a gynogenetic fate.

We found that for few hybrid embryos, the foreign sperm had triggered all necessary events for zygotic development, except for centrosome reformation. These embryos were not able to divide and eventually died. From this, we concluded that the paternally provided centrioles are necessary for zygotic development in *Mesorhabditis*, reinforcing the idea that *Mesorhabditis* males are an investment in centrioles for the reproduction of asexual females [5]. That centrioles are involved in a species barrier is a surprising finding. Interestingly, in vitro reconstitution of centrosomes from distantly related species has been successful in several cases, for instance after injection of human centrioles into *Xenopus* or starfish egg extracts [34]. To our knowledge, the involvement of centrosomes in hybrid breakdown has not been described so far. Whether this is a common feature of hybrid breakdown that has been overlooked or a specificity of these autopseudogamous species is an exciting question.

## Conclusion

Although *Mesorhabditis* species with a biased sex ratio had been described in the past, specimens had not been maintained in culture nor frozen down. We have here reestablished a culture collection of species (and identified two new species) and confirmed reproductive isolation between species. We found that a biased sex ratio is systematically associated with a pseudogamous reproductive strategy, in which absence of male-male competition leads to a drastic reduction of the male reproductive apparatus. Our collection of sexual and pseudo-sexual species thus offers an ideal framework for further exploration of the origin and consequence of transition to asexuality.

## Methods

### Nematode isolation and culture

*Mesorhabditis* nematodes live in rotting vegetal matter (Table S1) and are morphologically distinct from other terrestrial nematodes (see Supplementary Material). Strains were founded by a single isolated gravid female that produced at least one male progeny, allowing for further strain propagation. In the pseudogamous populations, 10–20 single females were isolated. Of these single females, a few did not lay eggs and were presumably not mated. The fertile females often produced few progeny (generally ca. 10–50), and in a given population about half produced only females (exact numbers were not recorded). We hypothesize that such females were old, because we previously showed that F1 males are produced early in the reproductive cycle. Eventually, a single fertile pseudogamous line was kept per sample.

Strains were cultured as for *C. elegans* [35] and were grown at 20 °C. All strains were frozen in DMSO 3.6%, Trehalose 3%, followed by washes in L-Glutamine 0.03% after thawing.

### Sequencing and phylogenetic tree reconstruction

ITS2 and 28S rDNA loci were amplified by PCR on single worm using the following primers: ITS2_forward 5’GCTGCGTTATTTAACGAATTGCARAC-3′, ITS2_reverse 5′-CACTTTCAAGCAACCCGAC-3′ (reverse), 28s_forward 5’AGCGGAGGAAAAGAAACTA-3′ and 28S_reverse 5’ACGATCGATTTGCACGTCAG-3′. Sequences are accessible on Genbank, accession number MT710227-MT710292. Using MEGA7 (Molecular Evolutionary Genetics Analysis version 7.0 for bigger datasets [36]), we obtained sequence alignment (with Muscle); pair-wise distance calculation and phylogenetic tree reconstruction (with UGPMA method). The evolutionary distances were computed using the Maximum Composite Likelihood method, and are expressed in number of base substitutions per site (out of 1595 positions). All ambiguous positions were removed for each sequence pair (pairwise deletion option).

### Crosses between strains

Four to 10 young females, at the L4 stage, were isolated on a single plate. The next day, we searched for the presence of eggs on plates, to exclude those plates containing non-virgin females. Virgin females were then crossed with 2 to 5 males from another strain. After 3 days, the results of the crosses were analysed. When F1s were produced, we waited one more week to monitor the production of F2s. For most strain pairs, the crosses were performed multiple times.

### Morphological characterization

Adult males were immobilized in  50 mM sodium azide and mounted on 2% agar pads between a slide and a coverslip. Images were taken using a Zeiss Axioskop or a Zeiss Axioimager A1, equipped with a digital Kappa camera DX4–285 FW (Figure S2). To measure male spicule, ray and sperm size, males were cut-open in a watch glass containing Egg Buffer (118 mM NaCl, 2 mM MgCl_2_, 25 mM Hepes (pH 7.2), 48 mM KCl, 2 mM CaCl_2_). The carcasses were then mounted directly between slide and coverslip, without an agar pad. This allowed for the flattening of the male tail and observation from the ventral side. This mounting procedure also resulted in the dispersion of mature sperm cells. Images were taken as described above, using a 100X DIC lens.

To measure body size, worms were first synchronized as described in [5]. We then waited until the animals reached the L4 stage, as monitored by the presence of a vulval invagination in females and of the fully formed spicules in males. L4 animals were then mounted in 50 mM sodium azide on an agar pad and imaged using a 10X lens. Body size was measured using the ImageJ Freehand drawing tool.

### Time-lapse DIC recording and embryo recovery

One-cell embryos were recorded as described in [5]. After the reformation of the parental pronuclei, embryos were recovered and placed onto fresh plates. After 4 days, we could clearly distinguish males and females in the dissecting microscope.

### Immunostaining and MitoTracker red labeling

Fixation of embryos was performed as described in [5], using a freeze-cracking method. For stainings, we used a mouse anti-tubulin antibody (1/200; DM1A; Sigma-Aldrich) and Hoechst (33,342; Sigma-Aldrich). Donkey anti-mouse secondary antibody Dylight 488 was used at 1/1000 (Jackson ImmunoResearch Laboratories).

For sperm staining, males were placed in a 100 µL drop of 100 µM Mitotracker Red CMXRos (Invitrogen) on a agar plate for 2 h in the dark. Next, they were allowed to recover on a fresh plate overnight. Crosses with stained males and virgin females were then performed as described above. After 48 h of mating, females were mounted between a slide and a coverslip on an agar pad. Image acquisition were as described above.

### Sex ratios

In well-growing pseudogamous populations, each female tends to lay its eggs at a given position on the plate, without moving around. For sex ratio analysis, the resulting piles of eggs were collected and placed onto fresh plates. After 4 to 6 days, adult males and females were counted. From one pile of eggs, we recovered between 100 and 300 animals. We used two piles for each measurement. At least 3 sex ratio measurements were performed per strain, on different days.

### Statistics

We tested how variation in reproductive traits (sex ratio, body size, spicule size, ray size and sperm area) is explained by reproductive mode. For that, we used generalized linear mixed models (GLMM) (R MASS package http://www.stats.ox.ac.uk/pub/MASS4). We assumed a Gaussian distribution for ray size, spicule size, body size and sperm area and a quasibinomial distribution for the sex ratio (which is a proportion). For explanatory variables, we modeled the reproductive mode as the fixed effect and the species as the random effect. For the body size model, we added sex and the interaction between sex and reproductive mode as fixed effects.

## Supplementary information

**Additional file 1:** Supplementary text: Determination of species and rationale for raising two new species

**Additional file 2: Table S1. Collection of** ***Mesorhabditis*** **strains and species used in this study (XLSX 31 kb)**

**Additional file 3: Table S2. Matrix of pair-wise distance (XLS 71 kb)**

**Additional file 4: Table S3. Crosses between pseudogamous** ***Mesorhabditis*** **strains.** The table shows the result of crosses between males (top) and females (left) of various strain pairs. The outcome is color-coded: blue for viable F2 progenies, black for no embryo production, light grey for production of dead embryos and light blue for mixed results, including the production of sterile F1s for some strain pairs. N represents the number of independent crosses that have been performed per strain pair.

**Additional file 5: Table S4. Measures of the sex ratio and morphology in different** ***Mesorhabditis*** **strains.** Measures of sex ratio, body size, spicule size, ray size and sperm area are shown on separate sheets.

**Additional file 6: Table S5. Development of amphimictic and gynogenetic embryos for 5 different species.** Results on *M. belari* JU2817 are from (Grosmaire et al., 2019). This table does not reflect the proportion of amphimictic versus gynogenetic embryos. For the resulting sex ratio, see Fig. 3.

**Additional file 7: Figure S1. Phylogeny of** ***Mesorhabditis*** **strains.** The evolutionary history was inferred using the UPGMA method. The optimal tree with the sum of branch length = 0.66366318 is shown. Bootstrap values (100 replicates) are shown next to the branches. The tree is drawn to scale, with branch lengths in the same units as those of the evolutionary distances used to infer the phylogenetic tree. All ambiguous positions were removed for each sequence pair (pairwise deletion option). There were a total of 1357 positions in the final dataset.

**Additional file 8: Figure S2. Male tails of the pseudogamous** ***Mesorhabditis*** **species.** Nomarski micrographs, in lateral view. The figure provides two examples for each species. Anterior is to the left on the left panels and on the right to the right side, except for panels I, J where both animals are oriented with their head to the left; the ventral side is down. All panels are at the same scale. Scale bar: 10 μm.

**Additional file 9: Figure S3. Labelling of the female spermatheca after crosses with MitoTracker Red labelled males.** One representative gonad of a *M. monhystera* female, after co-culture for 48 h with labelled males of *M. monhystera* (upper panel) or *M. belari* (lower panel). DIC images are overlayed with the fluorescent images shown in red. Arrowheads point toward the spermatheca. Immature oocytes are on the left, and the uterus is on the right of the spermatheca. A fertilized egg is visible in the uterus in the upper panel.

**Additional file 10: Figure S4. Phenotypes of hybrid embryos between different species pairs.** Still images from DIC recordings showing hybrid embryos from crosses of *M. belari* and *M. okuensis*, or *M. belari* and *M. microbursaris*. All embryos are blocked before the first cell division, after pronuclear envelope breakdown. Scale bar is 10 μm.

**Additional file 11: Movie S1.** Wild-type gynogenetic embryo of *M. belari* JU2817

**Additional file 12: Movie S2.** Wild-type amphimictic embryo of *M. belari* JU2817

**Additional file: 13 Movie S3.** Wild-type gynogenetic embryo of *M. simplex* JU2864

**Additional file 14: Movie S4.**. Wild-type amphimictic embryo of *M. simplex* JU2864

**Additional file 15: Movie S5.** Hybrid embryo from a cross between *M. simplex* JU2864 female and *M. belari* JU2817 male, with defects in eggshell formation, leading to egg explosion.

**Additional file 16: Movie S6.** Hybrid embryo from a cross between *M. simplex* JU2864 female and *M. belari* JU2817 male, showing normal early cellular events until nuclear envelop breakdown

## Data Availability

The sequences generated and analysed during the current study are available on Genbank via accession number MT710227-MT710292.

## Abbreviations

DIC: Differential interference contrast
DMSO: Dimethyl sulfoxide
DNA: Deoxyribonucleic acid
F1: First filial generation
F2: Second filial generation
ITS2: Internal transcribed spacer
PCR: Polymerase chain reaction
rDNA: Ribosomal deoxyribonucleic acid
RNA: Ribonucleic acid
S phase: Synthesis phase
UGPMA: Unweighted pair group method with arithmetic mean
