# Supplementary material for: Diversification and hybrid incompatibility in auto-pseudogamous species of Mesorhabditis nematodes
Source: BMC Evol Biol. 2020 Aug 18;20:105. doi: 10.1186/s12862-020-01665-w (PMC7433073; doi:10.1186/s12862-020-01665-w)
Supplement: Supplementary file 1 — Additional file 1: Supplementary text: Determination of species and rationale for raising two new species [file 12862_2020_1665_MOESM1_ESM.pdf]

Determination of species and rationale for raising two new species

The *Mesorhabditis* was a subgenus of *Rhabditis* by (Osche, 1952) and raised to the genus level by Dougherty 1953.

Mesorhabditis nematodes live in rotting vegetal matter (humus, leaf litter, rotting fruits, stems and leaves; Table S1) and are morphologically distinct from other terrestrial nematodes because of i) a posterior vulva, ii) small body size, iii) dark intestine (Barrière and Félix, 2014). Strains were sampled as described in (Barrière and Félix, 2014). Strains were founded by a single isolated gravid female that produced at least on male progeny, allowing for further strain propagation. We isolated 69 new strains: 40 from samples coming from Europe (12 from France), 18 from Asia, 4 from Africa, 3 from North America, 2 from South America and 2 from Oceania (see Table S1). 47/69 strains showed a highly biased sex ratio with very few males and were categorized as pseudogamous strains. In Europe, the pseudogamous species such as *M. belari* or *M. monhystera* were common in leaf litter in woods, while *M. spiculigera* appeared in richer environment such as compost, rotting fruits and stems (Table S1).

We also obtained from colleagues 3 previously isolated strains: *M. microbursaris* PS1179 (isolated by Lynn Carta), *M. longespiculosa* DF5017 (by Walter Sudhaus), and *M. spiculigera* AF72 (by Andras Fodor).

In Europe, the pseudogamous species such as *M. belari* or *M. monhystera* were common in leaf litter in woods, while *M. spiculigera* appeared in richer environment such as compost, rotting fruits and stems (Table S1).

Sudhaus surveyed in (Sudhaus 1978) the *Mesorhabditis* species where males occur at high frequency (close to 50%). He calls this group of species the *Spiculigera* group in (Sudhaus 2011). The strain *M. longespiculosa* DF5017 was isolated and first described in (Sudhaus 1978). The strain JU764 was determined as *M. spiculigera* (Steiner 1936) by W. Sudhaus. We isolated many more isolates of *M. spiculigera* but from our samples of soil and rotting vegetation, we did not isolate further species in this group.

Regarding species with rare males and a vestigial bursa, which constitute the *Monhystera* group, Sudhaus (2011) catalogued 15 species and indicates that a comparative study of this group is lacking. Note some species (but not *M. belari*) in the *Monhystera* group of *Mesorhabditis* in (Sudhaus 2011) are classified in *Bursilla* by (Andrássy 1984).

We first delineated species using crosses and molecular tags in ribosomal DNA (see Figure S1 and Figure 1). We then aimed to assign names catalogued in (Sudhaus 2011), based on morphological indications from the original descriptions. None of the described species in this group has been kept in culture, thus we compared our cultures to the morphological description, with some insight from their geographical origin. We aimed to minimize the number of new names to be assigned.

The most distinctive character that can be compared with prior descriptions is the length and shape of the male tail. In the pseudogamous species, the caudal papillae (rays) are highly variable within a given strain and often differ on the left and right sides of a given individual. We thus focused instead on length and shape of the male tail. Taking into account a published species thus required that the initial description includes a description of the male tail (Figure S2). We could not use some descriptions where the male tail was not drawn.

Three species have been described to have a short male tail: *M. belari* (Nigon 1949), *M. paucipapillata* (Paetzold 1955) and *M. labiata* (Völk 1950). *M. labiata* was described to have four lips in the buccal region, which we never saw - therefore, we did not use this name.

We commonly found in Western Europe a species, represented by strain JU2817, with the morphology described by (Nigon 1949) for *Rhabditis* (now *Mesorhabditis*) *belari*. Nigon notes that the morphology of *M. belari* resembles that drawn by (Cobb 1893a) for "*monhystera*" where the male tail is very short but differs from the long tail drawn by (de Man 1927). We followed de Man, Nigon and (Sudhaus 2011) in assigning the species name *R. monhystera* to a species with a long male tail (see below) and determined the species including JU2817 to be

M. belari. The tail tip of animals of various isolates of this species is short, with a variable irregular shape, sometimes bifurcate (Figure S2A).

The species closest to *M. belari* from molecular tags (Figure 1) is represented by European strains JU2858, JU2890, JU3149 and JU3285. The males of this species display a short tail, with a slightly more elongated and pointed tip compared to *M. belari*. Some individual males display a more irregular tail shape, with a knob or bifurcate tip (Figure S2B). The pointed shape matches well the morphology of *M. paucipapillata* (Paetzold 1955), also described from Europe. We thus determined the species represented by JU2858 as *M. paucipapillata*.

Among our isolated species with a short tail, three species remained, which all also include at least some individual males with a bifurcate tail tip: i) a species isolated from California (JU2864), New Zealand (JU3229 and JU3248) and China (JU3344 and JU3346); ii) a species isolated in Italy (JU2902) and South India (JU3211), presenting most often the characteristic bifurcate tail and iii) a species from Cameroon represented by strains JU3143, JU3147 and JU3148. The species *M. simplex* (Cobb 1893b) has been described from Australia, with a minimal morphological description: no drawing of individuals of either sex and no comparison to other species (Cobb 1893b). To avoid unnecessary new names, we assigned this name to the species isolated from California, New Zealand and China. Its tail tip is generally simpler than that of the other species in this group, but may be bifurcate in some individuals (Figure S2C, right panel). We name in the section below the two other species *M. bifurcata* n. sp. and *M. okuensis* n. sp., respectively.

Among species with a long male tail (Figure S2F-K), the most common species in Europe is represented by strains JU2855 and others. In addition to this geographic pattern, its long tail with an intermediate narrowing region (Figure S2F) and the particularly short spicules (Figure 4D) match the original description of *Rhabditis monhystera* Bütschli (Frankfurt / Main), considering the drawing by (de Man 1927) (strain from Netherlands). We thus determine the species represented by strains JU2855 and others as *M. monhystera*.

The species represented by PS1179 displays a particularly long male tail and a near absence of fan (Figure S2G). We determined it to be *M. microbursaris* (Steiner 1926) based on this description showing a tail with the longest aspect in the drawing.

M. franseni (Fuchs 1933), *M. littoralis* (Yeates 1969) and *M. vernalis* (Andrassy 1982) were considered synonyms by (Zeidan and Geraert 1990); *M. franseni* was in addition considered a synonym of *M. microbursaris* by (Andrássy 1984). Here we attribute *M. franseni* originally found in Europe by Fuchs (Germany) to the species represented by strain JU2870 that we found in Vienna, a species displaying a long male tail (but not as long as *M. microbursaris*) with a slight narrowing down at a posterior position relative to the rays, almost no fan and few rays of very reduced size (Figure S2H). We use *M. vernalis* for the species represented by JU2847 (from Brazil) and strains from China and Japan, and *M. littoralis* for JU2848. Their male tail resembles that of *M. franseni* with a narrowing down after the most posterior rays (Figure S2H-J). For the last remaining species found in South India and Sao Tome, with a long pointed tail and highly reduced rays (Figure S2K), we use the species name *M. cranganorensis* (Khera 1968) - this species was described from females collected in South India and no males were found.

Species descriptions

The electronic edition of this article conforms to the requirements of the amended International Code of Zoological Nomenclature (ICZN), and hence the new names contained herein are available under that Code from the electronic edition of this article. This published work and the nomenclatural acts it contains have been registered in ZooBank, the online registration system for the ICZN. The ZooBank LSIDs (Life Science Identifiers) can be resolved and the associated information viewed through any standard web browser by appending the LSID to the prefix ‘‘http://zoobank.org/’’. The LSID for this publication is:

urn:lsid:zoobank.org:pub:A6F4DB4E-50C8-4DB6-85BC-2D21DD80AE19. The electronic edition of this work was published in a journal with an ISSN.

Mesorhabditis bifurcata Launay, Félix, Dieng and Delattre sp. nov.
Zoobank identifier urn:lsid:zoobank.org:act:66909C76-8FD8-4248-91CF-81E3C686EBB9
The type isolate by present designation is JU2902, deposited at the Caenorhabditis Genetics Center. The species is delineated and diagnosed by the fertile cross with the type isolate JU2902 in both cross directions, yielding fertile hybrid females and males that are interfertile and cross-fertile with their parent strains. Males occur at low frequency. This species differs by LSU and ITS2 DNA sequences from all species listed in Table S1 of the present manuscript. Note that these ribosomal DNA sequences may vary within the species. The type isolate was collected from rotting pseudostem of *Musa basjoo*, sampled on 12 Dec 2015 in the Botanical Garden of the University of Florence, Italy. The species was also found from compost in Bangalore, India. The male tail is short and generally ends with a bifurcate tip (Figure S2). The mouth is endowed with six lips. The species is named after the bifurcate tip of its male tail.

Mesorhabditis okuensis Launay, Félix, Dieng and Delattre sp. nov.
Zoobank identifier urn:lsid:zoobank.org:act:191A39D4-7A5D-42A9-A6B4-8F712569405B
The type isolate by present designation is JU3143, deposited at the Caenorhabditis Genetics Center. The species is delineated and diagnosed by the fertile cross with the type isolate JU3143 in both cross directions, yielding fertile hybrid females and males that are interfertile and cross-fertile with their parent strains. Males occur at low frequency. This species differs by LSU and ITS2 DNA sequences from all species listed in Table S1 of the present manuscript. Note that these ribosomal DNA sequences may vary within the species. The type isolate was collected from rotting wood sampled in Oku Mountain, Cameroon in May 2016. The male tail is short, similar or slightly longer than that of *M. belari* (strain JU2817) and may end with a bifurcate tip (Figure S2). The mouth is endowed with six lips. The species is named for its place of isolation.

Andrassy, I. (1982). Six new species of the suborder Rhabditina (Nematoda). Revue de Nématologie **5**: 39-50.

Andrássy, I. (1984). Klasse Nematoda. Stuttgart, Gustav Fischer Verlag.

Barrière, A. and Félix, M.-A. (2014). Isolation of C. elegans and related nematodes. *WormBook Online Rev. C Elegans Biol.* 1–19.

Cobb, N. A. (1893a). Nematodes, mostly Australian and Fijian. Department of Agriculture New South Wales, Miscellaneous Publications **13**: 1-59.

Cobb, N. A. (1893b). Plant diseases and their remedies. Diseases of the sugar-cane. Agricultural Gazette of New South Wales **4**: 777-833.

de Man, J. G. (1927). Das Männchen der Rhabditis monhystera Bütschli. Zoologischer Anzeiger **70**: 51-57.

Dougherty, E. C. (1953). The genera of the subfamily Rhabditinae Micoletzky, 1922 (Nematoda). Thapar Commemoration Volume: 69-76.

Fuchs, G. (1933). Einige Nematoden bei *Scolytus scolytus* F. Capita Zoologica **4**: 1-45.

Khera, S. (1968). Nematodes from the banks of still and running waters. IV. Description of a new subgenus of Rhabditis and a new species from India (Subfamily Rhabditinae). J. zool. Soc. India **20**: 38-41.

Nigon, V. (1949). Modalités de la reproduction et déterminisme du sexe chez quelques nématodes libres. Ann. des Sc. Nat., Zool. **11**: 1-132.

Osche, G. (1952). Systematik und Phylogenie der Gattung Rhabditis (Nematoda). Zoologische Jahrbücher (Systematik) **81**: 190-280.

Paetzold, D. (1955). Untersuchungen an freilebenden nematoden der Salzwiese bei Aseleben. Wissenschaftliche Zeitschrift der Martin-Luther-Universität Halle-Wittenberg **4**: 1057-1090.

Steiner, G. (1926). Parasitic nemas on peanuts in South Africa. Centralblatt Bakteriologie **67**: 351-365.

Steiner, G. (1936). Opuscula miscellanea nematologica, III. Proceedings of the Helminthological Society of Washington **3**: 16-22.

Sudhaus, W. (1978). Systematik, Phylogenie und Ökologie der holzbewohnenden Nematoden-Gruppe *Rhabditis (Mesorhabditis)* und das Problem "geschlechtsbezogener" Artdifferenzierung. Zool Jb Syst Bd **105**: 399-461.

Sudhaus, W. (2011). Phylogenetic systematisation and catalogue of paraphyletic "Rhabditidae" (Secernentea, Nematoda). J. Nem. Morph. Syst. **14**: 113-178.

Völk, J. (1950). Die Nematoden der Regenwürmer und aasbesuchenden Käfer. Zool. Jb. Syst. **79**: 1-70.

Yeates, G. W. (1969). Three new Rhabditida (Nematoda) from New Zealand dune sands. Nematologica **15**: 115-128.

Zeidan, A. B., Geraert, E. (1990). Free-living nematodes from Sudan. Nematologica **35**: 277-304.
