# Supplementary material for: Diversification and hybrid incompatibility in auto-pseudogamous species of Mesorhabditis nematodes
Source: BMC Evol Biol. 2020 Aug 18;20:105. doi: 10.1186/s12862-020-01665-w (PMC7433073; doi:10.1186/s12862-020-01665-w)
Supplement: Supplementary file 6 — Additional file 6: Table S5. Development of amphimictic and gynogenetic embryos for 5 different species. Results on M. belari JU2817 are from (Grosmaire et al., 2019). This table does not reflect the proportion of amphimictic versus gynogenetic embryos. For the resulting sex ratio, see Fig. 3. [file 12862_2020_1665_MOESM6_ESM.docx]

**Table S5: Development of amphimictic and gynogenetic embryos for 5 different species**

Results on *M. belari* JU2817 are from (Grosmaire et al., 2019). This table does not reflect the proportion of amphimictic versus gynogenetic embryos. For the resulting sex ratio, see Figure 3.

| Species | Strain | Amphimictic develop as males | Amphimictic develop as females | Gynogenetic develop as males | Gynogenetic develop as females |
| --- | --- | --- | --- | --- | --- |
| *M. belari* | JU2817 | 30 | 0 | 0 | 85 |
| *M. paucipapillata* | JU3003 | 2 | 0 | 0 | 10 |
| *M. simplex* | JU2864 | 11 | 0 | 0 | 13 |
| *M. okuensis* | JU3143 | 0 | 0 | 0 | 10 |
| *M. bifurcata* | JU2902 | 0 | 0 | 0 | 7 |
| *M. monhystera* | JU2855 | 4 | 0 | 0 | 10 |
