# Supplementary figures and images for: Diversification and hybrid incompatibility in auto-pseudogamous species of Mesorhabditis nematodes
Source: BMC Evol Biol. 2020 Aug 18;20:105. doi: 10.1186/s12862-020-01665-w (PMC7433073; doi:10.1186/s12862-020-01665-w)

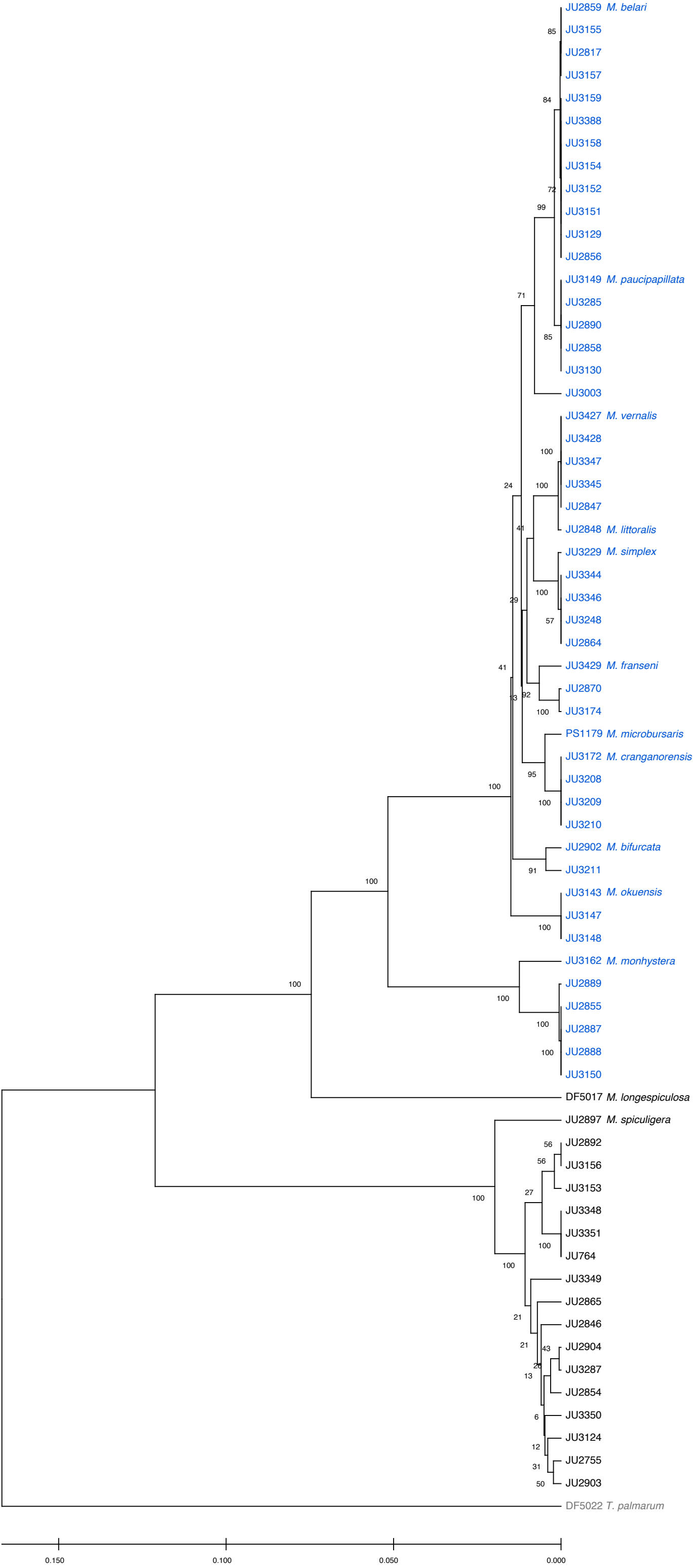

Figure S1

Supplement: Supplementary file 7 — Additional file 7: Figure S1. Phylogeny of Mesorhabditis strains. The evolutionary history was inferred using the UPGMA method. The optimal tree with the sum of branch length = 0.66366318 is shown. Bootstrap values (100 replicates) are shown next to the branches. The tree is drawn to scale, with branch lengths in the same units as those of the evolutionary distances used to infer the phylogenetic tree. All ambiguous positions were removed for each sequence pair (pairwise deletion option). There were a total of 1357 positions in the final dataset. [file 12862_2020_1665_MOESM7_ESM.pdf]

female *M. monhystera* x male *M. monhystera*

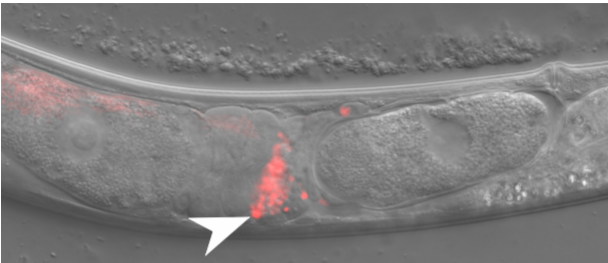

female *M. belari* x male *M. monhystera*

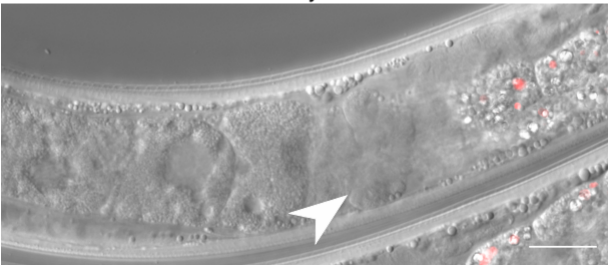

Figure S3

Supplement: Supplementary file 9 — Additional file 9: Figure S3. Labelling of the female spermatheca after crosses with MitoTracker Red labelled males. One representative gonad of a M. monhystera female, after co-culture for 48 h with labelled males of M. monhystera (upper panel) or M. belari (lower panel). DIC images are overlayed with the fluorescent images shown in red. Arrowheads point toward the spermatheca. Immature oocytes are on the left, and the uterus is on the right of the spermatheca. A fertilized egg is visible in the uterus in the upper panel. [file 12862_2020_1665_MOESM9_ESM.pdf]

*M. okuensis* JU3143 x *M. belari* JU2817

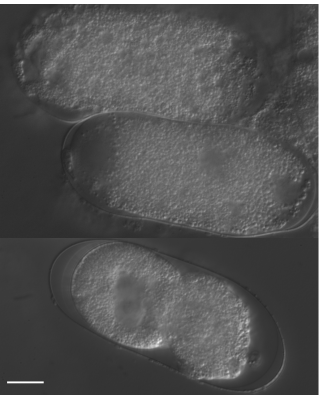

*M. okuensis* JU3147 x *M. belari* JU2817

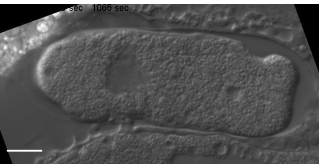

*M. microbursaris* PS1179 x *M. belari* JU2817

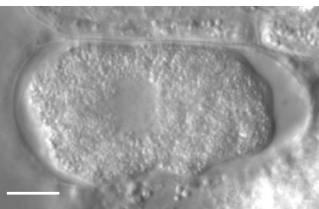

Figure S4

Supplement: Supplementary file 10 — Additional file 10: Figure S4. Phenotypes of hybrid embryos between different species pairs. Still images from DIC recordings showing hybrid embryos from crosses of M. belari and M. okuensis, or M. belari and M. microbursaris. All embryos are blocked before the first cell division, after pronuclear envelope breakdown. Scale bar is 10 μm. [file 12862_2020_1665_MOESM10_ESM.pdf]
