# Supplementary material for: Diversification and hybrid incompatibility in auto-pseudogamous species of Mesorhabditis nematodes
Source: BMC Evol Biol. 2020 Aug 18;20:105. doi: 10.1186/s12862-020-01665-w (PMC7433073; doi:10.1186/s12862-020-01665-w)
Supplement: Supplementary file 8 — Additional file 8: Figure S2. Male tails of the pseudogamous Mesorhabditis species. Nomarski micrographs, in lateral view. The figure provides two examples for each species. Anterior is to the left on the left panels and on the right to the right side, except for panels I, J where both animals are oriented with their head to the left; the ventral side is down. All panels are at the same scale. Scale bar: 10 μm. [file 12862_2020_1665_MOESM8_ESM.pdf]

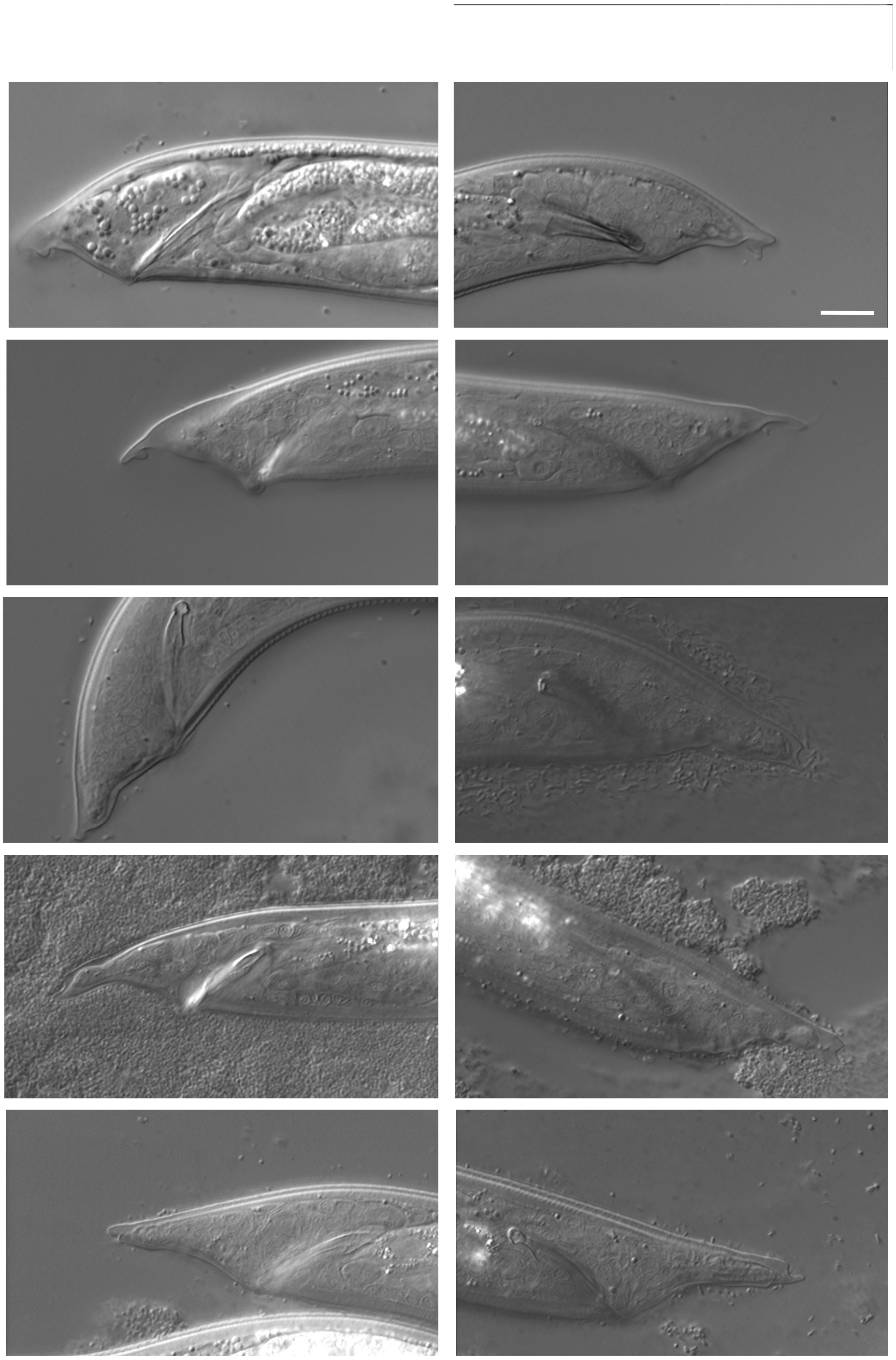

(A)
M. belari
JU2817

(B)
M. paucipapillata
JU2858

(C)
M. simplex
JU2864 and JU3248

(D)
M. bifurcata
JU2902

(E)
M. okuensis
JU3143

Figure S2

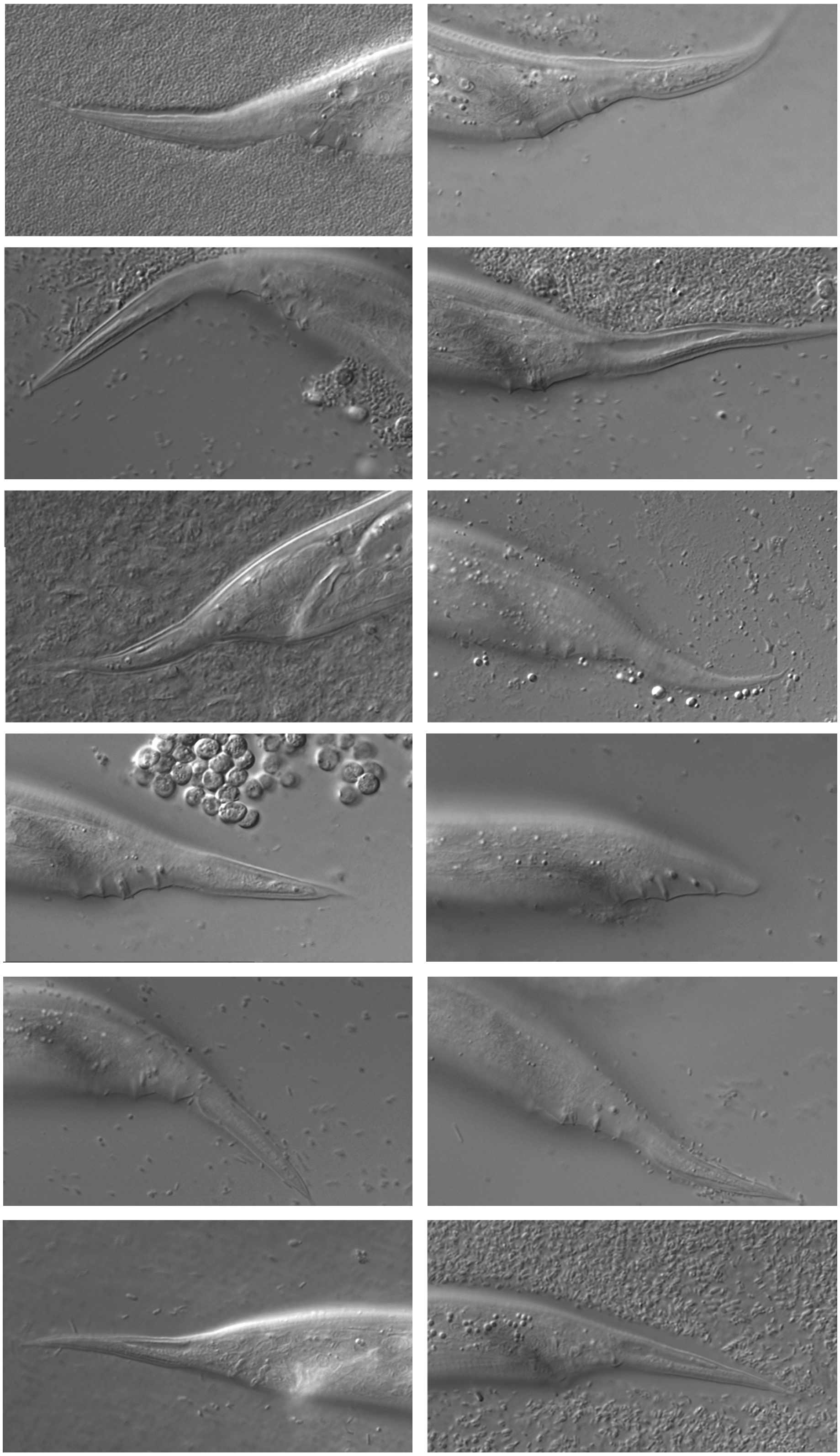

(F)
M. monhystera
JU3162 and JU2855

(G)
M. microbursaris
PS1179

(H)
M. franseni
JU3174

(I)
M. vernalis
JU2847

(J)
M. littoralis
JU2848

(K)
M. cranganorensis
JU3210 and JU3172

Figure S2 (continued)
